# Tmoato industrial derivatives: mallardo reaction and residual allergenicity

**DOI:** 10.1186/2045-7022-1-S1-P19

**Published:** 2011-08-12

**Authors:** Valerio Pravettoni, Laura Primavesi, Marta Piantanida, Oreste V Brenna, Larua Farioli, Joseph Scibilia, Ambra Mascheri, Elide Anna Pastorello

**Affiliations:** 1 Foundation IRCCS Cà Granda Ospedale Maggiore Policlinico, Clinical Allergy and Immunology Unit, Milan, Italy; 2 University of MIlan, Dipartimento di Scienze e Tecnologie Alimentari e Microbiologiche (DISTAM), Milan, Italy; 3 Niguarda Cà Granda Hospital, Laboratory Medicine, Milan, Italy; 4 Niguarda Cà Granda Hospital, Allergology and Immunology, Milan, Italy

## Background

tomato is the third most cultivated crop around the world, 4.64% of which derived from Italy, where tomato production is mainly focused on industrial derivatives (about 85%).In a previous study we demonstrated that, differently from other lipid transfer protein (LTP) containing fruits, in tomatoes LTP is contained in peel, pulp and seeds.

## Aim of the study

To investigate the influence of technological processing, usually applied in tomato industry, on the allergenicity of the end product in respect to fresh tomato fruit. We analyzed twenty-two processed tomato derivatives purchased on Italian market, and two tomato extracts obtained from whole tomatoes and chemically peeled tomatoes.

## Methods

The thermal damage index in all tomato derivatives was determined chromatographically by detecting furosine level, which allowed us to divide the commercial products in low, medium and highly thermally damaged. SDS-PAGE and immunoblotting on samples of these three groups was performed. We used the patients’ sera from our previous study after obtaining informed consent. Five patients had a documented positive history of severe allergic reactions to tomato, fresh or household cooked or industrially processed, and were exclusively reacting to tomato LTP. Other five patients experienced oral allergy syndrome (OAS) grade I-II when eating fresh tomatoes and were sufferings from birch pollinosis and not reacting to tomato LTP.

## Results

In LTP-positive patients, no statistical difference between chemically peeled and raw extracts was detected by means of skin tests. Any grade of thermal damage (low, medium or high furosine index) induced a significant reduction in tomato allergenicity in birch pollen-positive LTP-negative patients, while none of the investigated technological processes reduced the IgE-binding to tomato LTP in LTP-positive patients.

## Conclusions

LTP-positive patients with clinical symptoms to tomatoes should avoid commercial tomato products even if subjected to high thermal treatment.

